# Advancements in targeted and immunotherapy strategies for glioma: toward precision treatment

**DOI:** 10.3389/fimmu.2024.1537013

**Published:** 2025-01-14

**Authors:** Guangyuan Gong, Lang Jiang, Jing Zhou, Yuanchao Su

**Affiliations:** ^1^ Department of Intensive Care Medicine, Jiangsu Provincial People’s Hospital Chongqing Hospital (Qijiang District People’s Hospital), Chongqing, China; ^2^ Department of Thoracic Surgery, Jiangsu Provincial People’s Hospital Chongqing Hospital (Qijiang District People’s Hospital), Chongqing, China; ^3^ Department of Emergency Medicine, Jiangsu Provincial People’s Hospital Chongqing Hospital (Qijiang District People’s Hospital), Chongqing, China

**Keywords:** high-grade glioma, immunotherapy, targeted therapy, molecular biology, treatment

## Abstract

In recent years, significant breakthroughs have been made in cancer therapy, particularly with the development of molecular targeted therapies and immunotherapies, owing to advances in tumor molecular biology and molecular immunology. High-grade gliomas (HGGs), characterized by their high malignancy, remain challenging to treat despite standard treatment regimens, including surgery, radiotherapy, chemotherapy, and tumor treating fields (TTF). These therapies provide limited efficacy, highlighting the need for novel treatment strategies. Molecular targeted therapies and immunotherapy have emerged as promising avenues for improving treatment outcomes in high-grade gliomas. This review explores the current status and recent advancements in targeted and immunotherapeutic approaches for high-grade gliomas.

## 1 Introduction

Gliomas, the most common primary central nervous system tumors, originate from glial cells and are classified into grades I-IV by the WHO, with grades III and IV being high-grade gliomas (1). Grade IV gliomas, including glioblastomas (GBM), are the most prevalent, comprising 46.1% of gliomas (2). High-grade gliomas encompass various subtypes such as anaplastic astrocytomas and anaplastic oligodendrogliomas, each with distinct molecular and histological characteristics. Additionally, glioblastomas are further categorized into newly diagnosed glioblastoma (nGBM) and recurrent glioblastoma multiforme (rGBM), distinguishing initial diagnoses from cases of tumor recurrence.

HGGs are highly malignant, prone to rapid recurrence, and resistant to conventional therapies like surgery, radiotherapy, and chemotherapy, with a poor prognosis and a 5-year survival rate of just 5.5% for GBM (2). The immune microenvironment plays crucial roles in disease’s progression and influences the effectiveness of treatments (3–5). This poor prognosis is partly due to the immunosuppressive tumor microenvironment, which includes tumor-associated macrophages (TAMs), myeloid-derived suppressor cells (MDSCs), regulatory T cells (Tregs), and immune checkpoint molecules such as PD-1/PD-L1, all contributing to immune evasion and resistance to therapies (6–8). These factors present significant challenges in developing effective treatments. In addition to the immunosuppressive microenvironment, the blood-brain barrier (BBB) significantly impedes the delivery of therapeutic agents to gliomas (9). Recent advancements have focused on strategies to enhance the permeability of the BBB or utilize alternative delivery mechanisms to improve the efficacy of immunotherapies in HGGs (10).

In recent years, molecular targeted therapies and immunotherapies have emerged as promising strategies to address these challenges (11–15). Targeted therapies aimed at specific genetic alterations, such as IDH1 mutations, EGFR amplification, and PTEN loss, are currently under investigation (6). Immune checkpoint inhibitors, including PD-1/PD-L1 and CTLA-4 blockers, have shown potential in preclinical and clinical trials, though their clinical application is hindered by the complex glioma immune microenvironment. This review aims to summarize the current status and recent advancements in molecular targeted and immunotherapeutic strategies for High-grade glioma (HGG), highlighting their potential to improve patient outcomes and the challenges that remain in their clinical implementation.

## 2 Molecular targeted therapy

Molecular targeted therapy designs drugs to address molecular abnormalities within tumors (16, 17), inhibiting growth and metastasis. Due to the heterogeneity of HGG, molecular targeted therapies have become a focal point in clinical research (**Table 1**). Recent studies in genetic profiling have highlighted the critical role of personalized medicine in tailoring treatments for HGG. By identifying genetic alterations such as EGFR mutations, IDH1 mutations, and PTEN deletions, clinicians can select targeted therapies that offer the highest potential benefit for individual patients.

**Table 1:** Clinical trials of combined therapy for HGG.

Type	Clinical phase	Treatment target	Combined treatment	PFS(m)	OS(m)	Reference
nGBM	I	EGFR	Nimotuzumab/RT/TMZ	10	15.9	(26)
Nonenhancing	I	IDH1	Ivosidenib	13.6	NR	(44)
nGBM	II	VEGF	Bevacizumab/Hypofractionated RT	7.6	NR	(19)
nGBM	II	EGFR	Nimotuzumab/RT/TMZ	11.9	24.5	(29)
UM-*MGMT*	II	mTOR	Paxalisib	8.4	17.7	(34)
*BRAF*-*V600E*	II	BRAF/MEK	Trametinib/Dabrafenib	3.8	17.6	(40)
nDIPG	III	EGFR	Nimotuzumab/RT	5.8	9.4	(31)

### 2.1 Vascular endothelial growth factor inhibitors

Angiogenesis plays a key role in HGG, with VEGF inhibitors like bevacizumab targeting VEGF to block tumor growth. The AVAglio and RTOG0825 phase III trials showed that BEV modestly extended progression-free survival (PFS) in newly diagnosed glioblastoma (nGBM), reduced corticosteroid use, and improved quality of life, but had no significant effect on overall survival (OS) (18, 19). Follow-up from RTOG0825 revealed increased neurocognitive decline and quality-of-life deterioration in BEV-treated patients, raising concerns about its neurotoxicity (20). Safety studies suggest that BEV does not interfere with standard treatments or amplify radiotherapy toxicity (21). A phase II trial by Wirsching et al. (22) found that BEV combined with hypofractionated radiotherapy (40 Gy/15 F) significantly extended PFS (7.6 months) in elderly nGBM patients, though it did not improve OS, highlighting the need to balance BEV’s benefits with its neurotoxic effects. Anlotinib, a multi-target tyrosine kinase inhibitor, targets VEGFR, PDGFR, FGFR, and c-Kit, inhibiting angiogenesis. It has a low incidence of adverse effects and significantly prolongs PFS in recurrent HGG patients (23). A 2021 phase II pre-trial at the ASCO meeting showed that anlotinib was safe and tolerable in nGBM patients, with a median OS of 17.4 months and one-year PFS and OS rates of 84.0% and 100.0%, respectively, supporting further investigation (24).

### 2.2 Epidermal growth factor receptor inhibitors

EGFR mutations, particularly EGFR, are common in GBM and serve as key therapeutic targets in HGG (25). Nimotuzumab, a humanized monoclonal antibody targeting EGFR, was tested in Chinese nGBM patients, with Wang et al. (26) reporting good safety and tolerability, a median PFS of 10.0 months, and OS of 15.9 months in 26 patients. These results, comparable to standard therapy, were not linked to EGFR expression, consistent with a phase III trial (27). Further studies suggested that Akt and mTORC1 signaling could predict nimotuzumab efficacy in GBM (28). In a phase II trial by Du et al. (29), nimotuzumab extended PFS to 11.9 months and OS to 24.5 months in 36 nGBM patients, with no survival difference between MGMT promoter methylation-positive and negative groups. Nimotuzumab also showed clinical efficacy in pediatric HGG, including diffuse intrinsic pontine glioma (DIPG) (30). A phase III trial across Germany, Italy, and Russia found that combining nimotuzumab with radiotherapy in pediatric DIPG patients was as effective as chemotherapy but with fewer toxicities and better safety profiles (31).

Although EGFR and EGFR mutations are prevalent in HGG, EGFR-targeting tyrosine kinase inhibitors (TKIs) and antibodies have not significantly improved survival in glioma patients (32). Greenall et al. (33) found that most EGFR-targeting antibodies were ineffective at neutralizing EGFR, while panitumumab could neutralize both wild-type EGFR and EGFR, showing strong anti-tumor effects *in vitro* and *in vivo*. This suggests panitumumab as a promising candidate for future clinical trials in glioma patients with EGFR mutations.

### 2.3 Mammalian target of Rapamycin inhibitors

mTOR, a critical target in the PI3K-AKT pathway, regulates cell proliferation, differentiation, and angiogenesis. Paxalisib, a small-molecule inhibitor targeting PI3K/AKT/mTOR, crosses the blood-brain barrier. A phase II trial (NCT03522298) at the 2022 ASCO Annual Meeting assessed paxalisib in MGMT promoter methylation-negative nGBM patients. The study found 60 mg to be the maximum tolerated dose, with PFS and OS of 8.4 and 17.7 months, respectively, showing improved efficacy over standard treatment (34). Paxalisib has received FDA fast track designation for GBM, with a confirmatory trial (NCT03970447) ongoing.

### 2.4 Mitogen-activated protein kinase pathway inhibitors

The MAPK cascade, particularly the RAS-RAF-MEK-ERK pathway, regulates cell survival, proliferation, and differentiation. The BRAF V600E mutation, present in ~6% of GBM cases, leads to persistent activation of this pathway, driving tumorigenesis. Epithelioid GBM, with BRAF V600E mutations in up to 50% of cases, has a poor prognosis, with an OS of 10 months (35, 36). Xia et al. (37) showed that vemurafenib, a BRAF inhibitor, combined with radiotherapy, reduced proliferation and increased apoptosis in BRAF V600E-mutant cells. Preliminary results from the VE-BASKET study indicated vemurafenib’s anti-tumor activity in GBM patients with the mutation (38). A case report of an epithelioid GBM patient treated with dabrafenib showed disease stabilization, but progression occurred after 10 months, with death at 16 months (39). A phase II trial of dabrafenib and trametinib (MEK inhibitor) in recurrent gliomas with BRAF V600E mutations enrolled 45 patients, with 15 showing objective responses, including 3 complete responses. Median PFS and OS were 3.8 and 17.6 months, respectively, suggesting promising effects, but further studies are needed (40).

### 2.5 IDH1 targeted therapies

IDH1 mutations are present in over 70% of WHO grade II and III gliomas, as well as in GBM derived from these low-grade lesions. These mutations are associated with a better prognosis compared to IDH wild-type gliomas of the same grade (41). In the fifth edition of the WHO glioma classification, IDH-mutant diffuse astrocytomas are now classified as a single entity, including grades 2, 3, and 4, and are no longer subdivided into diffuse astrocytomas, anaplastic astrocytomas, and GBM. IDH-mutant GBM is now termed IDH-mutant astrocytoma (1). Ivosidenib, an oral inhibitor of mutant IDH1, has been used in treating cholangiocarcinoma and chondrosarcoma (42, 43). A phase I trial by Mellinghoff et al. (44) in 66 patients with IDH1-mutant advanced GBM showed that daily 500 mg doses of ivosidenib had a favorable safety profile and reduced the volume and growth rate of non-enhancing tumors on MRI. Although many targeted therapies show limited efficacy in clinical trials, small-scale studies suggest promising preliminary results. With advances in next-generation sequencing, a deeper understanding of glioma molecular phenotypes and pathways may enhance the future utility of targeted therapies.

## 3 Immunotherapy

Immunotherapy, which utilizes the body’s immune system to target and eliminate tumor cells, is increasingly applied to solid tumors (45–47). However, its use in HGG faces significant challenges, including the blood-brain barrier, a highly suppressed tumor immune microenvironment, and immune resistance. Addressing these obstacles is a critical focus in glioma immunotherapy research. Glioma immunotherapy can be categorized into tumor vaccines, oncolytic viruses, immune checkpoint inhibitors, and chimeric antigen receptor T-cell (CAR-T) immunotherapy (**Table 2**).

**Table 2:** Clinical trials of combined therapy for HGG.

Type	Clinical phase	Treatment target	Combined treatment	PFS(m)	OS(m)	Reference
GBM	I	Dendritic cell vaccine	TMZ/CMVpp65-Vac	25.3	41.1	(53)
DIPG	I	Oncolytic virus	DNX-2401/RT	10.7	17.8	(58)
Her2+ rGBM	I	CAR-T	CAR-T	3.5	24.5	(73)
nGBM	II	Peptide vaccine	SurVaxM	11.4	25.9	(51)
GBM	II	Dendritic cell vaccine	Glioblastoma stem cell-like antigen	7.7	13.7	(54)
nHGG	II	GMCI	ADV-TK	8.1	17.1	(59)
GBM	II	ICI	Navurizumab	4.1	7.3	(69)
nGBM	II	ICI	Atezolizumab/RT/TMZ	10.6	19	(72)

### 3.1 Tumor vaccines

Tumor vaccines hold promise for treating HGG. Rindopepimut, targeting EGFR, initially showed benefits in PFS and OS in a phase II trial with rGBM patients treated with temozolomide (TMZ). However, a phase III trial with 745 patients found no significant difference in median OS, leading to early termination (48, 49). A phase II trial indicated that combining rindopepimut with bevacizumab (BEV) may improve PFS in rGBM patients (50).

SurVaxM, targeting survivin, demonstrated superior efficacy in a phase II trial for nGBM, with median PFS and OS of 11.4 and 25.9 months, respectively, surpassing standard treatments. A phase III trial is ongoing to confirm these findings (51). DCVax-L, an autologous dendritic cell vaccine, significantly extended OS in both nGBM and rGBM patients, with median OS of 19.3 months for nGBM and 13.2 months for rGBM patients, compared to 16.5 and 7.8 months in the control groups, respectively (52).

Cytomegalovirus (CMV) antigens, expressed in over 90% of GBM cases but absent in normal brain tissue, present a novel therapeutic target. Batich et al.’s phase I trial using a dendritic cell vaccine targeting CMV pp65 showed promising results, with median PFS and OS of 25.3 and 41.1 months, respectively, and some patients progression-free for over 7 years (53). Yao et al.’s phase II trial using dendritic cell vaccines loaded with GBM stem cell-like antigens demonstrated improved survival, with B7-H4-low expressing patients showing significantly better OS, indicating B7-H4 as a new target for glioma immunotherapy (54). Neoantigen vaccines like NeoVax, tested in a phase Ib trial for nGBM, showed an OS of 16.8 months, supporting their ability to activate T-cell responses (55). Platten et al.’s phase I trial with an IDH1-targeting vaccine (IDH1-vac) led to a 3-year PFS of 63% and OS of 84%, marking a significant improvement in patient outcomes (56). ERC1671, combining whole inactivated tumor cells and tumor cell lysates, showed significant effects in rGBM patients, particularly those naive to or resistant to BEV, with an average OS of 328 days and a correlation between peripheral blood CD4+ T lymphocyte counts and survival (57). These results highlight the potential of tumor vaccines as a key component of glioma immunotherapy, warranting further investigation.

### 3.2 Oncolytic viruses

Oncolytic viruses eliminate tumor cells through two mechanisms: (1) direct cytotoxicity via infection and replication within tumor cells, and (2) immune activation, converting tumors from an immune “cold” to a “hot” state. Recombinant oncolytic polio/rhinovirus (PVSRIPO), targeting the CD155 receptor on tumor cells and antigen-presenting cells (APCs), induces tumor lysis and activates immune responses. In a phase I trial of 61 rGBM patients, intratumoral PVSRIPO injection improved survival, with 21% surviving beyond 3 years, and the longest survival reaching 70 months (57). PVSRIPO has received “Breakthrough Therapy” designation by the FDA, and a phase II trial is ongoing. Although primarily studied in adult HGG, its use in Diffuse Intrinsic Pontine Glioma (DIPG) is limited.

Similarly, the oncolytic adenovirus DNX-2401, administered intratumorally, promotes immune infiltration and tumor responses. In a phase I trial of 11 pediatric DIPG patients, DNX-2401 followed by radiation therapy resulted in tumor shrinkage or stabilization, with a median PFS of 10.7 months and median OS of 17.8 months (58). Gene-mediated cytotoxic immunotherapy (GMCI), using a replication-deficient adenovirus to deliver the herpes simplex virus thymidine kinase (HSV-TK) gene, activates prodrugs like valganciclovir to induce cytotoxicity. In a phase II trial for HGG, the GMCI group had a median OS of 17.1 months, compared to 13.5 months in the standard treatment group, with the greatest benefit seen in patients with minimal residual tumor post-resection (59). GMCI has shown safety and efficacy in adults, with a phase I trial in pediatric HGG confirming its safety for children, supporting further studies (60).

### 3.3 Immune checkpoint inhibitors

PD-1/PD-L1 is a key immune checkpoint that enables tumor cells to evade immune surveillance (61, 62). However, clinical trials of PD-1/PD-L1 monoclonal antibodies in glioblastoma have had limited success, highlighting the need to understand resistance mechanisms. The presence of TAMs, MDSCs, and Tregs within the tumor microenvironment has been shown to interfere with the efficacy of immune checkpoint inhibitors (63). For instance, TAMs can express PD-L1 themselves, further contributing to the suppression of T cell activity (64). MDSCs inhibit T cell receptor signaling, reducing the effectiveness of PD-1 blockade (65). Additionally, Tregs maintain an immunosuppressive environment by expressing CTLA-4, which can negate the benefits of PD-1 inhibitors (66).

The CheckMate 498 trial found that nivolumab (PD-1 monoclonal antibody) did not improve OS in MGMT promoter-methylation-negative GBM patients, while CheckMate 548 showed no improvement in PFS in MGMT promoter-methylation-positive GBM patients, although OS is still under evaluation (67, 68). Neoadjuvant PD-1 therapy has shown promise in rGBM, with a phase II trial by Schalper et al. demonstrating that nivolumab before and after surgery altered the tumor immune microenvironment and improved outcomes, with median PFS of 4.1 months and OS of 7.3 months (69). Pembrolizumab, another PD-1 monoclonal antibody, showed limited efficacy as monotherapy in rGBM due to the immunosuppressive tumor environment. A phase I trial combining pembrolizumab with stereotactic radiosurgery and bevacizumab demonstrated safety, but a phase II trial combining pembrolizumab and BEV failed to improve survival in rGBM (70). However, neoadjuvant pembrolizumab followed by adjuvant treatment significantly improved OS and PFS in rGBM patients compared to adjuvant-only therapy, with enhanced immune responses, such as T-cell clonal expansion and reduced PD-1 expression on peripheral T-cells (71). Atezolizumab (PD-L1 inhibitor) showed moderate efficacy in a phase II trial, with median OS of 19 months in nGBM patients, particularly those with MGMT-methylation (72).

### 3.4 CAR-T therapy

CAR-T therapy involves genetically modifying T cells to express receptors targeting tumor cell antigens, leading to tumor destruction. In GBM, common targets include HER2 and IL-13Rα2. In a Phase I trial by Ahmed et al. (73), anti-HER2 CAR-T therapy in rGBM patients showed a median OS of 11.1 months post-infusion and 24.5 months from diagnosis, suggesting a survival benefit. Brown et al. (74) reported a multi-focal GBM patient treated with resection followed by intravenous IL-13Rα2 CAR-T infusion, leading to lesion shrinkage and disease stabilization for 7.5 months, indicating preliminary efficacy in HGG treatment.

A novel target for GBM CAR-T therapy is disialoganglioside (GD2), overexpressed on GBM stem cells. In a Phase I trial, eight GD2-positive rGBM patients received fourth-generation GD2-specific CAR-T cells (4SCAR-T), showing good safety, tolerability, and a median OS of 10 months post-infusion. GD2-specific CAR-T cells also induced antigen loss and immune activation in the tumor microenvironment, highlighting potential. Larger trials are needed to confirm these findings (75). Although CAR-T therapy for HGG has mainly been explored in small Phase I/II trials, current results support further investigation in larger cohorts. Despite promising early outcomes, challenges remain in the clinical application of immune therapies for HGG. However, a deeper understanding of glioma mechanisms and immune principles may offer solutions to immune resistance, potentially leading to longer survival for HGG patients.

Recent advancements have developed innovative strategies to enhance the efficacy of CAR-T cell therapy by overcoming the BBB. For instance, genetically engineered CAR-neutrophils derived from human pluripotent stem cells demonstrate improved BBB permeability and targeted delivery of tumor-responsive nanodrugs to GBM cells, thereby increasing therapeutic specificity and reducing off-target effects (9). Additionally, iPSC-derived BBB models have proven valuable for evaluating CAR-T cell extravasation and cytotoxicity against GBM, revealing significant differences among CAR-T constructs in traversing the BBB and eliminating tumor cells, which aids in optimizing CAR-T design for better clinical outcomes (76). Furthermore, Chokshi et al. (77) reported that CAR-T cells targeting receptors such as ROBO1 have shown promising preclinical results by effectively navigating the BBB and significantly extending survival in recurrent GBM and other brain metastasis models. These approaches not only enhance the penetration of CAR-T cells across the BBB but also improve their anti-tumor efficacy, thereby expanding the clinical potential of CAR-T therapies in treating high-grade gliomas.

## 4 Conclusion

Immunotherapy for HGG, particularly glioblastoma, has advanced significantly, providing novel treatment options beyond conventional therapies. Oncolytic viruses, including PVSRIPO and DNX-2401, have shown promise by directly targeting tumor cells and activating immune responses, with early clinical trials indicating survival benefits. Gene-mediated cytotoxic immunotherapy (GMCI) has also improved survival in both adult and pediatric HGG patients.

Immune checkpoint inhibitors, particularly PD-1/PD-L1 inhibitors, have produced mixed results in GBM, though neoadjuvant use may enhance immune responses in the tumor microenvironment. Combining these inhibitors with radiation or bevacizumab may further improve efficacy. CAR-T therapies targeting antigens like HER2, IL-13Rα2, and GD2 have shown promising early clinical outcomes, suggesting potential for durable responses in GBM treatment.

Despite progress, challenges such as immune evasion and tumor heterogeneity remain. Moreover, high costs and the complexity of patient-specific treatments limit the accessibility of CAR-T therapies, particularly in resource-limited settings. Future research will focus on overcoming these obstacles, refining treatment strategies, and ultimately improving long-term survival for GBM patients.
